# CodY-mediated regulation of *Streptococcus pyogenes* exoproteins

**DOI:** 10.1186/1471-2180-12-114

**Published:** 2012-06-21

**Authors:** Emily J McDowell, Eduardo A Callegari, Horst Malke, Michael S Chaussee

**Affiliations:** 1 Division of Basic Biomedical Sciences, The Sanford School of Medicine of the University of South Dakota, Vermillion, SD, USA; 2 Department of Microbiology and Immunology, University of Oklahoma Health Sciences Center, Oklahoma City, OK, USA

## Abstract

****Background**:**

The production of *Streptococcus pyogenes* exoproteins, many of which contribute to virulence, is regulated in response to nutrient availability. CodY is a transcriptional regulator that controls gene expression in response to amino acid availability. The purpose of this study was to identify differences in the expression of streptococcal exoproteins associated with deletion of the *codY* gene.

****Results**:**

We compared the secreted proteins produced by wild-type *S. pyogenes* to a *codY* mutant in the post-exponential phase of growth. We used both one and two-dimensional gel electrophoresis to separate exoproteins. Proteins that were significantly different in abundance upon repeated analysis were identified with tandem mass spectrometry. The production of the secreted cysteine protease SpeB, a secreted chromosomally encoded nuclease (SdaB), and a putative adhesion factor (Spy49_0549) were more abundant in supernatant fluids obtained from the *codY* mutant. In addition, hyaluronidase (HylA), CAMP factor (Cfa), a prophage encoded nuclease (Spd-3), and an uncharacterized extracellular protein (Spy49_0015) were less abundant in supernatant fluids obtained from the *codY* mutant strain. Enzymatic assays showed greater DNase activity in culture supernatants isolated in the post-exponential phase of growth from the *codY* mutant strain compared to the wild-type strain. Because extracellular nucleases and proteases can influence biofilm formation, we also measured the ability of the strains to form biofilms during growth with both rich medium (Todd Hewitt yeast extract; THY) and chemically defined media (CDM). No difference was observed with rich media but with CDM the biofilms formed by the *codY* mutant strain had less biomass compared to the wild-type strain.

****Conclusions**:**

Overall, the results indicate that CodY alters the abundance of a select group of *S. pyogenes* exoproteins, including DNases, a protease, and hylauronidase, which together may alleviate starvation by promoting dissemination of the pathogen to nutrient rich environments and by hydrolysis of host macromolecules.

## **Background**

*Streptococcus pyogenes* is thought to be responsible for more than 500,000 deaths worldwide each year [1]. Pathogenesis involves several proteins localized to the extracellular environment. These secreted proteins, or exoproteins, can be experimentally defined as those present in culture supernatant fluids. Exoproteins have a variety of functions and due to their localization most, if not all, interact with host molecules. Some have immunomodulatory effects, such as superantigens, which disrupt the immune response to infection by non-specifically stimulating T lymphocytes [2]. Others are cytolysins, such streptolysins O (SLO) and S (SLS), and many are hydrolytic enzymes that degrade host macromolecules to generate catabolic substrates or to promote tissue invasion. Examples of the latter include, hyaluronidase (HylA), which is required for growth using hyaluronic acid as the sole carbon source [3]; a secreted protease, SpeB, which is thought to promote dissemination by degrading a variety of extracellular matrix proteins, as well streptococcal various adhesins [4-6] and other secreted virulence factors such as nucleases and streptokinase [7,8]. Proteolysis can also liberate peptides and amino acids for catabolism. In addition, secreted nucleases promote dissemination by degrading nucleic acids present in neutrophil extracellular entrapment, or NETs [9,10]. Finally, secreted proteases and secreted nucleases are also likely to work together to disperse *S. pyogenes* biofilms, which are composed of both proteins and extracellular DNA [11].

The regulation of exoprotein production is complex and involves a variety of transcriptional regulatory proteins, many of which are influenced by the availability of various metabolic substrates [12-14]. Because *S. pyogenes* is auxotrophic for most amino acids, the pathogen's ability to respond to amino acid depletion is likely to be critical for survival within the human host. The response involves both the *relA*-dependent pathway mediated by accumulation of (p)ppGpp [15] and a *relA*-independent pathway [16,17], mediated, at least in part, by the transcriptional regulator CodY [18]. CodY is present in the genomes of many low G + C Gram-positive bacteria and mediates changes in expression in response to the availability of amino acids [19,20]. The protein binds to intracellular branched amino acids, which increases its affinity for DNA binding, typically resulting in the repression of gene expression. When branched chain amino acids are depleted, DNA affinity decreases allowing the initiation of transcription. Although usually considered to be a repressor, CodY activates expression of acetate kinase [21] and *bsfF*, which is a small RNA in *B. subtilis*[22].

In *S. pyogenes*, CodY controls the expression of genes involved in the response to nutritional stress, including genes encoding exoproteins. The transcript levels of 34 genes were previously compared between a wild-type strain of *S. pyogenes* and a *codY* mutant derivative by using quantitative reverse transcriptase PCR (qRT-PCR) [18]. Eleven of the genes were predicted to encode secreted proteins. The expression of four of these genes (*grabsagAsdaB/mf-1*, and *speB*) was greater in the wild-type strain compared to the mutant strain, while the expression of the remaining seven was less (*ngaprtSsclscpAskaslospeH*). Subsequently, by using DNA microarrays, inactivation of *codY* in *S. pyogenes* was found to alter the transcription of approximately 17% of genes in the chromosome, including several that encoded exoproteins [23]. Together, the results indicate that CodY is a global regulator controlling the transcription of a variety of genes, including some encoding exoproteins, which are likely to influence host-pathogen interactions [18,23].

The purpose of this study was to compare the exoproteins of a wild-type strain of *S. pyogenes* to a *codY* mutant strain to identify potential differences derived either at the transcriptional or post-transcriptional level. The results confirmed, at the protein level, several differences in expression previously predicted by transcript analyses and identified additional exoproteins with altered abundance following the deletion of *codY*.

## **Results**

### **Analysis of exoproteins by SDS-PAGE**

**Figure 1:**
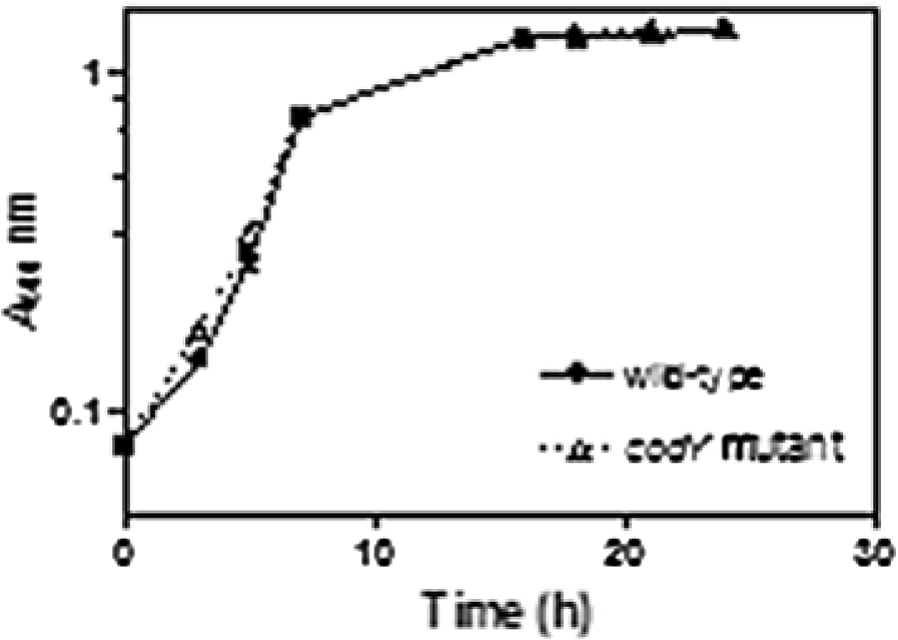
**Growth of wild-type and the*codY*mutant in CDM broth.** At various times during growth of the wild-type (·) and *codY* mutant (∆), the *A*_600_ of the cultures were determined.

**Figure 2:**
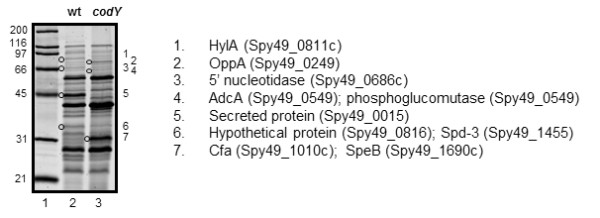
**CodY regulates exoprotein production.** SDS-PAGE gel analysis of 1) molecular weight standards and exoproteins isolated from 2) wild-type and 3) *codY* mutant strains of *S. pyogenes*. Open circles are adjacent to protein bands excised from the gel and numbers to the right of the gel designate the sample which was analyzed with by MS/MS. The protein with the highest score (and in some cases the protein with the 2^nd^ highest score) is indicated to the right of the gel image. The sizes (kDa) of molecular weight standards are shown to the left of the gel image. Additional information related to the MS/MS analyses is presented in Additional file 1: Table S1, Additional file 2: Table S2.

As an initial step to identify differences in exoprotein production between a *codY* mutant and a wild-type strain of *S. pyogenes*, the strains were grown to the stationary phase of growth and culture supernatant proteins (CSPs) were analysed by using SDS-PAGE gel electrophoresis. There was no difference in either the growth rate or growth yield of the two strains (Figure 1). Separation of CSPs by using SDS-PAGE showed several differences in the amounts of specific proteins (Figure 2). Seven protein bands were excised from the gel and analysed with tandem mass spectrometry (MS/MS; Additional file 1: Table S1, Additional file 2, Table S2). The results indicated that hyalurondidase (HylA; Spy49_0811c), which degrades hyaluronic acid present in the extracellular matrix of host tissue and the bacterial capsule, a 5’-nucleotidase (Spy49_0686c), a secreted protein with similarity to amidases (Spy49_0015), and a hypothetical protein possessing a type II secretion signal (Spy49_0816) were more abundant in the supernatant fluid obtained from the wild-type strain (Figure 2). In addition, the oligopeptide transport protein OppA (Spy49_0249) and a zinc binding protein AdcA (Spy49_0549) were less abundant in supernatant obtained from the wild-type strain (Figure 2). In some instances, MS/MS analyses of the excised protein bands detected peptides corresponding to more than one protein (Additional file 1: Table S1, Additional file 2: Table S2) indicating that SDS-PAGE was insufficient to completely separate the proteins. For example, protein band 7 (Figure 2, band 7) contained an equal number of peptides corresponding to the secreted protease SpeB (Spy49_1690c) and CAMP factor (Cfa; Spy49_1010c).

### **Analysis of exoproteins by two-dimensional gel electrophoresis (2-DE)**

**Figure 3:**
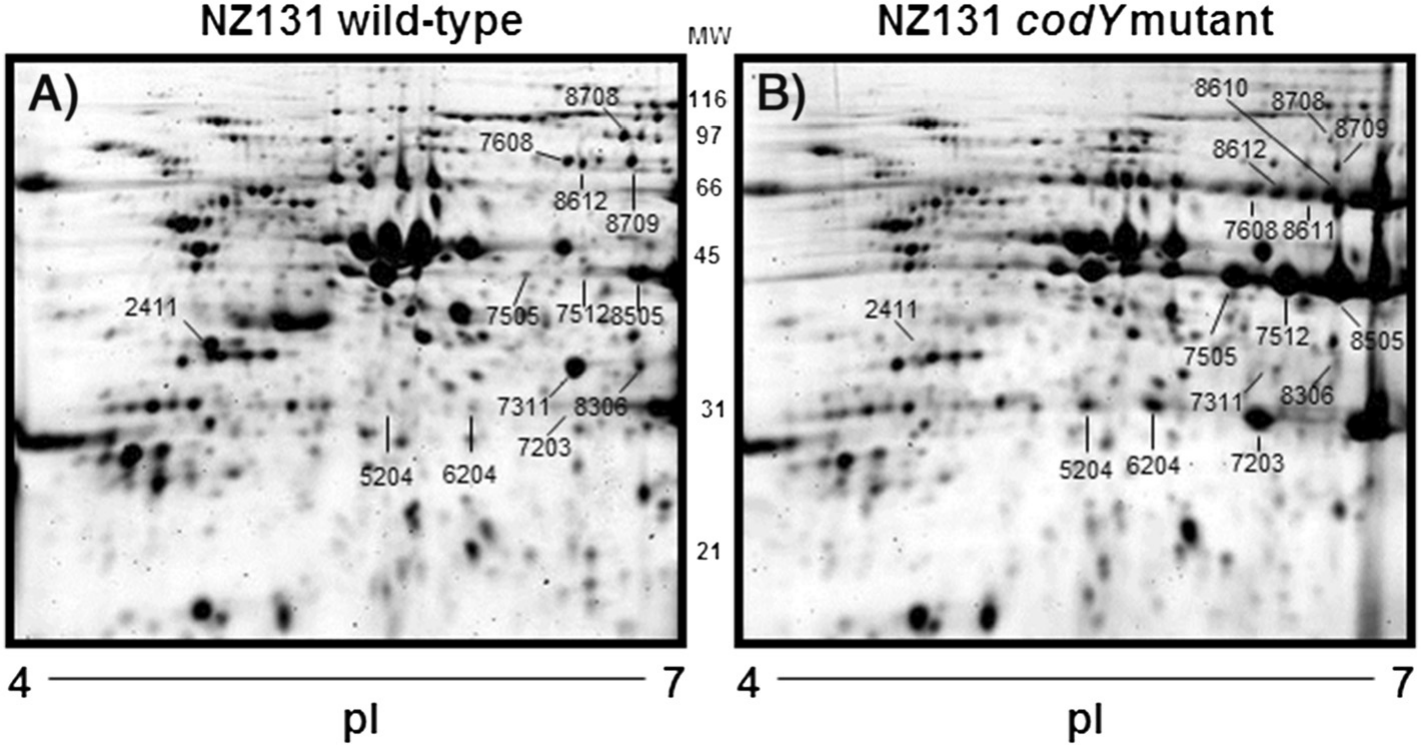
**2-D gel electrophoresis of culture supernatant proteins.** Proteins isolated from the **A**) wild-type and **B**) *codY* mutant strains were separated and numbered proteins were identified with MS/MS. The position of the spots is designated in both gel images, even if it the spot was not detected in CSPs obtained from one of the strains.

To better resolve the exoproteins 2-DE was used and images of representative gels are shown in Figure 3. The production of most exoproteins was not influenced by *codY* deletion, however several differences were noted (Table 1)**.** Differentially expressed proteins were excised from the gels and identified with MS/MS (Additional file 3: Table S3, Additional file 4: Table S4,**)**. In some instances proteins were differentially expressed in the representative gels shown in Figure 3 but not in the other biological replicates we identified only those proteins that were differentially expressed in all three biological replicates.

**Table 1:** **Protein spot abundance in wild-type and*codY*mutant strains**

**Spot No.**^***a***^	**Gene designation**^***b***^	**Name**	**Abundance**		**Fold difference**^**c**^
			**wt**	***codY***	
7311	1010c	Cfa	6,179	333	0.05
8306	1010c	Cfa	1,135	494	0.44
2411	1455	Spd-3	5,888	nd^***d***^	-
8505	1690c	SpeB	8,701	15,328	1.8
7505	1690c	SpeB	326	5,785	17.7
7512	1690c	SpeB	967	8,738	9.0
8612	0549	AdcA	235	3,889	16.5
7608	0549	AdcA	255	1,372	5.38
7203	1692c	SdaB	555	1,358	2.4
6204	1692c	SdaB	168	1,388	8.26
5204	1692c	SdaB	162	936	5.78
8709	0811c	HylA	1,253	739	0.59
8708	0811c	HylA	1,052	331	0.31
8610	0549	AdcA	nd	4,813	-
8611	0549	AdcA	nd	5,280	-

^**a**^ The number corresponds to the protein spot in Figure 3.

^***b***^ The open reading frame annotation based on the complete genome sequence of strain NZ131 (25).

^***c***^ The ratio *codY*/wt; a – indicated the protein was not detected in gels from one strain.

^***d***^ nd, not detected.

One of the most striking differences was the abundance of three positional variants of SpeB, which is a well-characterized cysteine protease that is secreted as a zymogen. Specifically, the spots designated 7505, 7512, and 8505 were 18-, 9-, and 2-fold more abundant, respectively in the *codY* mutant strain compared to the wild-type strain (Figure 3, Table 1). The results were consistent with previous reports indicating that *speB* transcripts were more abundant in the *codY* mutant strain when cultured with rich media, or blood [23,24].

### **Increased extracellular nuclease activity is associated with*codY*deletion**

**Figure 4:**
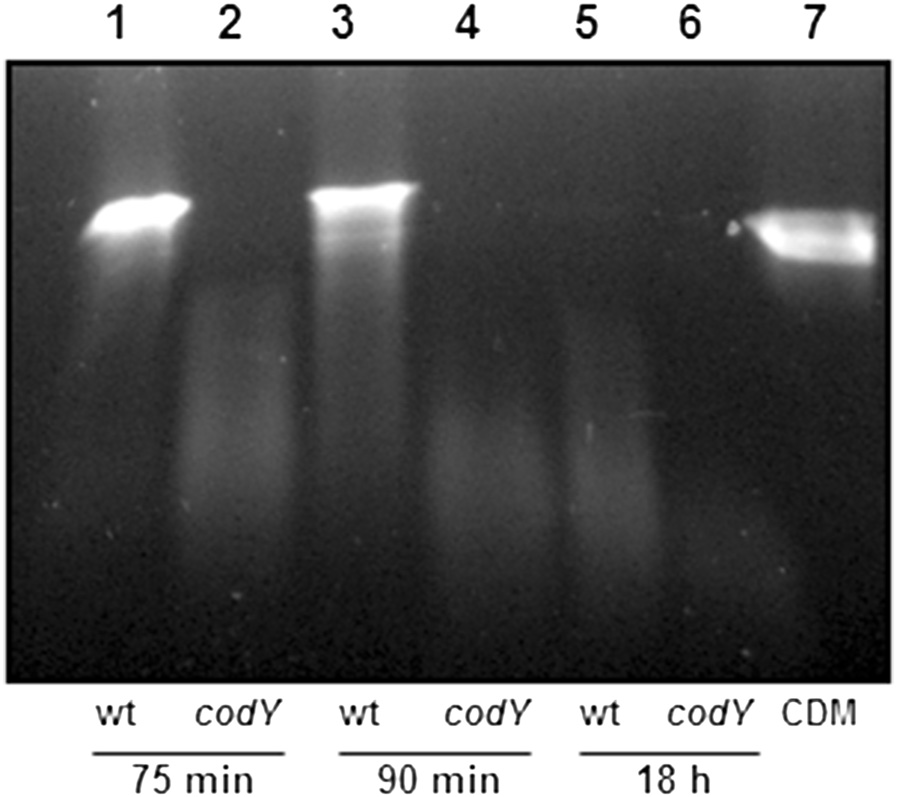
**CodY regulates extracellular nuclease activity.** Sterile CSPs were prepared from the wild-type and *codY* mutant strains grown under the same conditions that were used to analyze exoproteins by 2-DE. CSPs from the wild-type strain (lanes 1, 3, 5) and *codY* mutant (lanes 2, 4, 6) were incubated with DNA substrate for 75 min. (lanes 1,2); 90 min. (lanes 3,4); and 18 h (lanes 5, 6). As a control, sterile CDM broth was similarly incubated for 18 h with the DNA substrate (lane 7).

The genome of strain NZ131 encodes two secreted DNases. Streptodornase B (SdaB), also known as mitogenic factor 1 (Mf-1), is encoded within the bacterial chromosome. The other secreted nuclease, Spd-3, is encoded within a prophage [25]. Three SdaB isoforms (5204, 6204, and 7203) were 6-, 8-, and 2-fold more abundant in the *codY* mutant strain compared to the parental strain (Table 1, Figure 3). In contrast, Spd-3 (2411) was only detected in CSPs prepared from the wild-type strain (Figure 3, Table 1). Thus, the overall effect of *codY* deletion on extracellular nuclease activity remained unclear since SdaB was more abundant in the mutant but Spd-3 was less abundant. To address this issue, CSPs were isolated from the strains following 24 h culture with CDM and DNase activity was determined. The results showed that deletion of *codY* increased DNase activity (Figure 4).

### **Biofilm formation in CDM, but not rich medium, is influenced by*codY*deletion**

**Figure 5:**
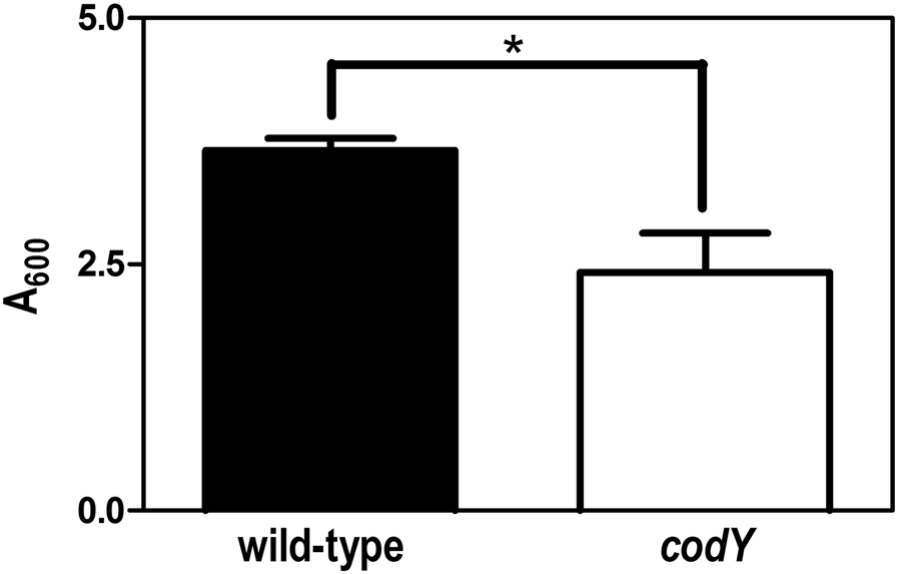
**Static biofilm formation is diminished in the*codY*mutant when cultured with CDM.** Biofilm formation was compared between the wild and *codY* mutant strains. The strains were cultured with CMD and static biofilm formation determined. An asterisk indicates the difference between the means is statistically significant (*P* < 0.05).

Static biofilms formed by *S. pyogenes* are dispersed by the addition of exogenous proteases and DNases, indicating the matrix is composed of both protein and DNA [11]. Based on differences in the production of the secreted protease SpeB and extracellular DNases between the two strains, and the influence of CodY on biofilm formation in related species [26-28], it was of interest to determine if deletion of *codY* altered biofilm formation of *S. pyogenes*. Static biofilm assays showed that deletion of *codY* consistently reduced biofilm formation compared to the parental strain when cultured with CDM (Figure 5); although both strains still formed biofilms. There was no difference in biofilm formation when the strains were cultured with THY medium (data not shown). Together the results indicate that CodY has a relatively minor, but reproducible, influence *S. pyogenes* biofilm formation under specific environmental conditions, perhaps due to changes in extracellular nuclease activity.

### **Deletion of*codY*affects the production of a putative zinc permease and CAMP factor**

A constellation of at least four variants of the uncharacterized protein AdcA (proteins spots 7608, 8612, 8611, and 8610) was more abundant in supernatants from the *codY* mutant strain (Figure 3, Table 1). A significant difference in *adcA* transcripts was not previously identified using DNA microarrays in either the exponential or stationary phases of growth [23]. The predicted 515 amino acid protein (Spy49_0549) has a putative signal peptide, a histidine rich motif, and is annotated as a zinc binding transporter [25]. It is part of the TroA superfamily, the members of which are involved in the transport of zinc into the cytoplasm. An AdcA orthologue in *Streptococcus pneumoniae* is a Zn^2+^ permease involved in the development of natural competence for DNA transformation [29,30] and the orthologue in *S. pneumoniae* and *S. gordonii* is required for both biofilm formation and competence [29-31]. We note that while AdcA was more abundant in the mutant strain, which did not form significant biofilms when cultured with CDM, the protein was detected in samples from the wild-type strain and thus production may have been sufficient to support biofilm production.

**Figure 6:**
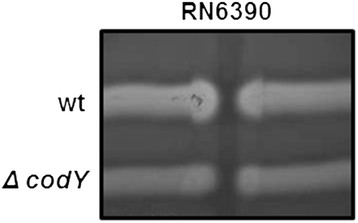
**Decreased Cfa activity in the*codY*mutant.***S. aureus* strain RN6390 was inoculated vertically on a blood-agar plate and wild and *codY* mutant strains of *S. pyogenes* were inoculated horizontally. Cfa activity is indicated by a wedge-shaped increase in hemolysis activity at the intersection of the two bacterial species.

In addition, two positional variants of CAMP factor (Cfa; 7311 and 8306) were less abundant in CSPs obtained from the *codY* mutant strain compared to the wild-type strain (Figure 3, Table 1). The results correlated with those obtained previously by measuring *cfa* transcripts [24]. Cfa is encoded as a 257 amino acid protein with a type II signal peptide. In a CAMP test, Cfa acts synergistically with the β hemolysin of *Staphylococcus aureus* to lyse erythrocytes. The CAMP test was used to compare Cfa activity between the two strains and the results showed that deletion of *codY* decreased Cfa activity (Figure 6). While it remains possible that potential differences in growth between the two strains on blood agar plates may contribute to the difference in CAMP factor activity the results are consistent with those obtained with proteomic analyses (Figure 3) and those obtained previously by measuring transcripts [23,24].

## **Discussion**

*S. pyogenes* exoproteins contribute substantially to interactions with the human host. Production is regulated by several, apparently redundant, transcriptional regulatory circuits working together to control expression. We used a proteomics approach to characterize the contribution of CodY to the regulation of *S. pyogenes* exoproteins. The purposes of this study were to clarify how previously identified differences in transcript levels between a wild-type and *codY* mutant strain are manifest at the protein level and to determine if *codY* deletion is associated with additional differences in the exoproteome due to post-transcriptional effects. The results confirmed, at the protein level, previously identified differences between the strains in the production of SpeB, Cfa, and SdaB. Moreover, additional exoproteins were discovered to be regulated by CodY, including the virulence associated secreted nuclease Spd-3, which is encoded by a prophage, a putative zinc binding transport protein AdcA, and HylA. Overall, the results contribute to defining the *S. pyogenes* exoproteome and the role CodY plays in determining its composition.

The proteolytically active form of SpeB can degrade several streptococcal exoproteins [7,32]. SpeB is secreted as a 40 kDa zymogen. It is subsequently converted to a 28 kDa proteolytically active form following a multi-step process involving intra- and inter-molecular SpeB cleavages and at least two peptidyl-prolyl, *cistrans* isomerases (RofA and PrsA; [32]). We harvested exoproteins by TCA/acetone precipitation prior to activation of the SpeB protease. Thus, under the conditions used in this study, only the zymogen form of SpeB was detected in the 2-DE gels and not the proteolytic form (Figure 3). In addition, no protease activity was detected in the culture supernatant samples (data not shown). Finally, the abundance of most exoproteins was similar between the two strains, despite the significant increase in SpeB zymogen production in the *codY* mutant strain, indicating that the exoproteins were not being degraded by SpeB in the mutant strain.

The production of two secreted nucleases was affected by *codY* deletion. The expression of SdaB was greater in the mutant strain, which is consistent with results previously obtained by using quantitative PCR during the exponential, but not stationary, phase of growth in rich media [18,23]. In contrast, the amount of the prophage-encoded Spd-3 protein was less in a *codY* mutant (Figure 3). This difference was not evident in a previous study in either the exponential or stationary phases of growth, respectively [23]. While this may indicate the involvement of post-transcriptional mechanisms in the regulation of Spd-3, it is noteworthy that the culture conditions used to identify differences in transcripts associated with *codY* inactivation differed from those used here to identify changes at the protein level, which may account for the differences among the studies.

DNase assays showed more activity in the *codY* mutant, which was consistent with the increase in SdaB production (Table 1, Figure 3). Previously, SdaB was reported to be the protein primarily responsible for extracellular DNase activity in a serotype M89 strain based on the absence of activity following *sdaB* inactivation [33].

The genome of strain NZ131 encodes four proteins with hyaluronidase motifs; two of these, Spy49_0785 and Spy49_1465c, are encoded by prophage and do not possess a signal peptide. Presumably, these proteins are released from the cell upon phage-induced lysis and degrade the hyaluronic capsule of *S. pyogenes*, which facilitates phage attachment and infection of streptococci [34,35]. Among the two chromosomally encoded proteins with hyaluronidase motifs, Spy49_1236c (designated Spy_1600 in strain SF370), which does not possess a signal peptide was recently discovered to have β-N-acetylgucosaminidase activity and not hyaluronidase activity [36]. Thus the only gene product possessing a signal peptide was the hyaluronidase protein (SpyM49_0811c) detected in supernatant preparations from the wild-type and *codY* mutant. Deletion of *codY* decreased the abundance of two positional variation of HylA, as detected in 2-DE gels, which correlated with results obtained with SDS-PAGE. Hyaluronidases are often thought of as spreading factors, facilitating dissemination of the pathogen; however, in murine models of *S. pyogenes* infection, HylA did not promote pathogen dissemination directly, but did increase the permeability of host tissue, which is likely to enhance toxin dissemination and thereby contribute to virulence [3].

## **Conclusions**

In summary, a proteomic approach was used to assess the role CodY plays in the regulation of *S. pyogenes* exoproteins. The results confirmed, at the protein level, that CodY regulates several well-studied exoproteins, including the SpeB protease and CAMP factor. In addition, we discovered new CodY regulated exoproteins including HylA. The results are important in understanding the roles various regulatory proteins play in controlling exoprotein production, which is intimately linked to the ability of the pathogen to adapt, and therefore survive, changing conditions encountered in its human host.

## **Methods**

### **Strains and culture conditions**

*S. pyogenes* strain NZ131 (serotype M49) and a *codY* mutant were previously described [18]. To construct the mutant strain, DNA flanking the *codY* open reading frame was amplified by PCR and cloned into pFW6 such that the fragments flanked the *aad9* gene, which confers resistance to spectinomycin [37]. After linearization, the recombinant plasmid (pFW6’aat-pncA) was used to transform NZ131. Transformants were obtained following deletion of the *codY* gene and substitution with the *aad9* gene [18]. Wild-type and *codY* mutant derivatives were inoculated from frozen stocks onto agar plates prepared with Todd-Hewitt supplemented with 0.2% yeast extract (THY) and incubated overnight at 37°C in 5% CO_2_. The bacteria were then suspended to an *A*_600_ of 0.08 in 40 ml chemically defined medium (CDM) [38] and incubated for 24 h at 37°C in 5% CO_2_.

### **Exoprotein isolation and separation**

Culture supernatant proteins were isolated from stationary phase cultures by trichloroacetic acid and acetone precipitation, as previously described [39]. Proteins were separated by sodium dodecyl sulfate polyacrylamide gel electrophoresis (SDS-PAGE) and two-dimensional gel electrophoresis (2-DE) using 10% acrylamide resolving gels, as previously described [40]. Gels were stained with SYPRO Ruby (BioRad, Hercules, Calif.) and imaged with the Typhoon 9410 variable mode imager using the 610BP 30 filter and 457 laser (GE Healthcare, Piscataway, NJ). Three independent protein isolations from both the wild-type and *codY* mutant strain were separated and the gels were analyzed with PDQuest software (Biorad). The abundance of proteins isolated with 2-DE was determined by summing the values of the pixels comprising the protein spot. The mean abundance of each protein was then determined from the three biological replicates obtained for each strain. Gels were normalized based on the sum of all protein spots detected in each sample. The CSPs were analysed for the presence of protease activity by using QuantiCleave Protease Assay Kit, as described by the manufacturer (Thermo Scientific, Rockford, Ill.). As a negative control, an NZ131 *speB* mutant strain was used. Standard curves were prepared with trypsin, as described by the manufacturer and purified SpeB protease was used as a positive control.

### **Protein identification**

Proteins of interest were excised from the SDS-PAGE gels with a robotic spot cutter (BioRad). The excised bands and spots were reduced with dithiothreitol (DTT; Sigma-Aldrich), alkylated with iodoacetamide (Sigma), and digested with sequencing grade trypsin (Promega) overnight at 37°C. The tryptic peptides were extracted by using 1% formic acid/2% acetonitrile in water followed by a second extraction using 50% acetonitrile/50% water. The extracts were concentrated with a SpeedVac centrifuge (Thermo Savant), dissolved in a solution of water/acetonitrile/formic acid (97/3/0.1%), and injected into a liquid chromatography instrument (nanoAcquity UPLC, Waters, Milford, MA). The peptides were desalted and concentrated online through an 180 μm X 20 mm, 5 μm Symmetry C18 nanoAcquity UPLC trap column (Waters) at a flow of 20 μL/min., with 99% solution A2 (water, 0.1% formic acid) and 1% solution B2 (100% acetonitrile, 0.1% formic acid) for 20 min. The peptides were separated online in the second dimension through a BEH130C18 1.7 μm, 100 μm X 100 mm nanoAcquity UPLC column. The standard solvent gradient used was: 0 to 2 min, 3% B2 isocratic; 2 to 40 min, 3–85% B2 linear, at a flow rate of 400 nL/min. for 60 min. The eluted ions were analyzed by one full precursor MS scan (400–1500 m/z) followed by four MS/MS scans of the most abundant ions detected in the precursor MS scan while operating under dynamic exclusion or direct data acquisition system. Spectra were obtained in the positive ion mode with a nano ESI-Q-Tof micro mass spectrometer (Micromass,UK), deconvoluted, and analyzed using the MassLynx software 4.1 (Micromass, UK). A peak list (PKL format) was generated to identify +1 or multiple charged precursor ions from the mass spectrometry data file. The instrument was calibrated in MS/MS mode using 500 fmole of (Glu^1^)-Fibrinopeptide B human with a RMS residual of 3.495 e^-3^ amu or 7.722 e^0^ ppm. Parent mass (MS) and fragment mass (MS/MS) peak ranges were 400–1500 Da and 65–1500 Da, respectively.

Mascot server v2.3.0 and Mascot Daemon Toolbox v2.3.0 (http://www.matrix-science.com, UK) in MS/MS ion search mode (local licenses) were applied to conduct peptide matches (peptide masses and sequence tags) and protein searches against NCBInr v20110707 (14605097 sequences; 4996850242 residues) using taxonomy filter S. *pyogenes* (24089 sequences, 6976687 residues). The following parameters were set for the search: carbamidomethyl (C) on cysteine was set as fixed; variable modifications included asparagine and glutamine deamidation and methionine oxidation. One missed cleavage was allowed; monoisotopic masses were counted; the precursor peptide mass tolerance was set at 2 Da; fragment mass tolerance was 0.3 Da. The MS/MS spectra were searched with MASCOT using a 95% confidence interval (C.I.% ) threshold (p < 0.05), with which a minimum score of 36 was used for peptide identification (identity or extensive homology). The protein redundancy that appeared at the database under different gi and accession numbers were limited to *S. pyogenes* with the first priority assigned to NZ131. All proteins identified were found within these domains.

### **Enzymatic activity assays**

To measure extracellular DNase activity, the wild-type and *codY* mutant strain were cultured for 24 h with CDM. Sterile CSPs were prepared exactly as was done for the protein analysis. CSPs were incubated for various times at 37°C with PCR-generated DNA from *S. pyogenes* and 1X New England Biolabs buffer 2. The CAMP test was done by inoculating *Staphylococcus aureus* RN6390 onto agar plates containing sheep blood and then inoculating the wild-type and *codY* mutant strains perpendicular to RN6390. The plates were incubated for 18 h at 30°C. Cfa activity is indicated by increased hemolysis at the intersection of *S. aureus* and CAMP factor-producing strains of *S. pyogenes*.

### **Biofilm assays**

Biofilm formation on polystyrene microtiter plates (Becton Dickinson, Lincoln Park, NJ) was done essentially as previously described [11]. Briefly, the strains were incubated with either CDM or THY for 24 h at 37°C in 5% CO_2_. Wells were washed three times with 200 μl of phosphate-buffered saline (PBS) and 200 μl of 0.1% (wt/vol) crystal violet was added to each well. After 30 min., the wells were washed twice with 200 μl of sterile deionized water to remove unbound crystal violet. The remaining crystal violet was dissolved in 200 μl of 95% ethanol and the absorbance was measured at 600 nm. Four wells were used for each strain and the average value determined. The experiment was repeated four times and the mean ± standard error of the mean is reported. The Student's *t*-test was used to determine if the mean values of biofilm formation differed between the strains.

## **Authors’ contributions**

EJM isolated and separated exoproteins, analyzed 2-DE gels, and drafted the manuscript. EAC identified proteins with mass spectrometry and co-authored the manuscript. HM constructed the strains and participated in the design of the study. MSC conceived of the study, and participated in its design and coordination and helped to draft the manuscript. All authors read and approved the final manuscript.

## Supplementary Material

Additional file 1 **Table S1.** Tandem mass spectrometry results of proteins excised from SDS-PAGE gel (Figure 2). Click here for file

Additional file 2 **Table S2. **Peptide characteristics used to identify proteins excised from SDS-PAGE gel (Figure 2). Click here for file

Additional file 3 **Table S3.** Tandem mass spectrometry results of proteins excised from 2-DE gel (Figure 3). Click here for file

Additional file 4 **Table S4.** Peptide characteristics used to identify proteins excised from 2-DE gel (Figure 3). Click here for file
