# Supplementary material for: CodY-mediated regulation of Streptococcus pyogenes exoproteins
Source: BMC Microbiol. 2012 Jun 21;12:114. doi: 10.1186/1471-2180-12-114 (PMC3438106; doi:10.1186/1471-2180-12-114)
Supplement: Additional file 1 — Table S1. Tandem mass spectrometry results of proteins excised from SDS-PAGE gel (Figure 2). [file 1471-2180-12-114-S1.docx]

| **Band #** | **Gene *^a^*** | **Name** | **Mol. mass** | **pI** | **Peptide matches*^b^*** | **Coverage*^c^* (%)** | **Score*^d^*** |
| --- | --- | --- | --- | --- | --- | --- | --- |
| 1 | 0811c | Extracellular hyaluronate lyase, HylA | 92579 | 6.2 | 9 | 14 | 153 |
| 2 | 0249 | Oligopeptide ABC transporter, OppA | 72101 | 5.4 | 20 | 35 | 557 |
| 3 | 0686c | 5-nucleotidase | 73312 | 5.7 | 8 | 16 | 294 |
| 4 | 0549 | Zinc-binding protein AdcA precursor | 58474 | 8.4 | 6 | 13 | 134 |
| 4 | 0970 | Phosphoglucomutase | 63217 | 4.8 | 7 | 15 | 121 |
| 5 | 0015 | Putative secreted protein | 41868 | 6.0 | 14 | 49 | 884 |
| 6 | 816 | Hypothetical protein | 34628 | 6.0 | 10 | 38 | 380 |
| 6 | 1455 | Streptodornase, Spd-3 | 30151 | 5.2 | 3 | 13 | 147 |
| 7 | 1690c | SpeB, streptococcal, cysteine protease | 43198 | 8.8 | 5 | 20 | 191 |
| 7 | 1010c | CAMP factor, Cfa | 28479 | 6.7 | 5 | 22 | 205 |

**Table S1. Tandem mass spectrometry results of proteins excised from SDS-PAGE gel (Fig. 2).**

^a^ The ORF designation is based on annotation of the NZ131 complete genome sequence (25).

^b^ The number of peptides matching the target protein.

^c^ The percentage of the protein sequence that was observed by MS/MS analysis.

^d^ The threshold was set up by Mascot server (hhtp://ww.matrixscience.com) based in Mowse algorithm at the significance level P> 0.05 for random hit, using 95% confidence interval, the minimum score of 36 was used for peptide identification (indicates identity or extensive homology).
