# Supplementary material for: CodY-mediated regulation of Streptococcus pyogenes exoproteins
Source: BMC Microbiol. 2012 Jun 21;12:114. doi: 10.1186/1471-2180-12-114 (PMC3438106; doi:10.1186/1471-2180-12-114)
Supplement: Additional file 2 — Table S2. Peptide characteristics used to identify proteins excised from SDS-PAGE gel (Figure 2). [file 1471-2180-12-114-S2.docx]

**Table S2. Peptide characteristics used to identify proteins excised from SDS-PAGE gel (Fig. 2).**

| Band # | Protein ID | Sequences | Parent mass | Charge State | Error (Da) |
| --- | --- | --- | --- | --- | --- |
| 1 | Extracellular hylauronate lyase (Spy_49_0811c) | YTAPIEK  AYTTIEQR  ADDKSPEAIK  QEFETYISK  KDDLEISDTIK  ELLSDTSVPVQK  TLVTQGNAFYNVYDNLK  TNYQQVGMTSLSDDAFVASK  LPGTTETEQKPLEGTPENIK | 820.4638  980.5234  1072.5548  1143.5798  1276.6704  1314.7372  1959.0282  2177.0332  2181.1666 | +2  +2  +2  +2  +2  +2  +2  +2  +3 | 0.0308  0.0308  0.0148  0.0351  1.0147  0.0342  0.0545  -0.9454  0.0501 |
| 2 | Oligopeptide ABC transporter, periplasmic oligopeptide-binding protein oppA (Spy_49_0249) | NVGIESVK  QSVEASLGK  ALNNKDFR  YVFVNDPK  LQDKPVTAK   QAIQFAFDR   LYPNDPTYK  AYQNGEVDFK   NFDKGEFTVAR   ASFQAQTAGQDAK  TTYSVLFPVNAK  LTYSDGSDPGSFYK  AYQNGEVDFKEVGVK  TYLDIMSPVGGGSVIQK  NYADNITYGMLTGDIR  KNYADNITYGMLTGDIR   HLTWNLNRTSFKNTK   AQAYLTDNAVDIPVVALGGTPR  EALTAEGVTFPVQLDYPVDQANAATVQEAQSFK | 844.5158  917.5474  976.5682  980.5590  998.6392  1097.6164  1109.6042  1169.5970  1282.6952  1321.7070  1338.7974  1535.7638  1682.9107  1780.0030  1831.9406  1960.0279  2181.1936  2240.3032  3536.9578 | +2  +2  +2  +2  +2  +2  +2  +2  +2  +2  +3  +2  +2  +3  +3  +3  +3  +3  +3 | 0.0504  0.0657  0.0592  0.0623  0.0632  1.0975  0.0649  -0.9223  -0.9194  0.0808  0.0791  0.0858  0.0955  0.0953  0.0996  0.0919  1.3641  0.1231  0.2267 |
| 3 | 5-nucleotidase (Spy_49_0686c) | IVQALSQGK   QANQDGTSIR   IGTATNSSTISK  TPSANVVAVAPGIK  NYVTSNLESSTK   TFPTVDFAMTNNGGIR IMTGQAPDPESTINDITK   IPNAGTAAQLGAYMDDAEIDFK | 942.6240  1088.5830  1178.7020  1322.8342  1341.7452  1756.9084  1946.0488  2326.2214 | +2  +2  +2  +2  +2  +2  +3  +3 | 0.0742  0.0620  0.0878  0.0785  0.1040  0.0994  0.1186  0.1427 |
| 4 | Zinc-binding protein adcA (Spy_49_0549) | LTTDVAGK  EIAQEINAH.-   AAVLSPLEGLTEK  K.YIYFEENASSK  GVIGNDGDVSMLMK  VVTTFYPVYEFTK | 802.5280 1023.5794  1326.8300  1349.7062  1466.7752  1592.8792 | +2  +2  +2  +2  +2  +2 | -0.9108  0.0810  0.0906  0.0923  0.1007  0.0666 |
| 4 | Phosphoglucomutase (Spy_49_0970) | SIVSTELVTK  QELLSMDEK  LIETKGEEAK   NLSGNQIGAIIAK  IANIETEINTFVG.-  TISVTLSGVDGAAEIK  NDGTISNLTTPPSNVLK | 1075.6882  1107.6124  1116.6760  1297.8184  1419.8064  1559.9296  1770.0414 | +2  +2  +2  +2  +2  +2  +2 | 0.0758  0.1007  0.0734  0.0831  0.0819  0.0890  0.1255 |
| 5 | Putative secreted protein Streptputative secreted protein (Spy_49_0015) | SISDVITR  QYIADHR  VMESNYSGK  NSELEELSK  TQQADLEVAK  VNLAIQLASAEDEK  AREEAAQQAASVEAAK   NNETSGYINALLNSK  GFEQEIQSLTNQIIAR  QAANQEAINTIAANMSAIK  SAYKNNETSGYINALLNSK  ASLLNQQAAAQQAAAEALAAQQAAQAK  QAANQEAINTIAANMSAIKENQNALR  ISALTAEQQSAQNQVNALQAQVSSLQAEQDK | 889.5484  901.5062  1029.5186  1047.5784  1101.6418  1499.8631  1628.9051  1636.8591  1846.0251  1974.0911  2086.1275  2635.4952  2799.4963  3297.8032 | +2  +2  +2  +2  +2  +2  +2  +3  +3  +3  +3  +3  +3  +3 | 0.0616  0.0657  0.0750  0.0701  0.0753  0.0800  0.0941  0.0540  0.0667  0.8767  0.0944  0.1276  0.1030  0.1595 |
| 6 | Hypothetical protein (Spy_49_816) | TNLSVAVK  QTAVISGSR  LPDDQVLDR  QIANGYEISK  LDSEVNNDTR  IKLDSEVNNDTR  LPDDQVLDRNYSSR  VEAVGMMDDSLSDIQYK  VTLQAVSADGTILASAIDPAK  SVPIRVEAVGMMDDSLSDIQYK  TVSLSSSQVSIEPSVVTVQLTTTKK.- | 830.5474  917.5594  1069.6008  1121.6356  1161.6002  1402.7804  1676.8969  1931.9434  2040.1916  2484.3070  2618.5408 | +2  +2  +2  +2  +2  +2  +3  +2  +2  +3  +3 | 0.0613  0.0664  0.0605  -0.9200  0.0741  0.0752  0.0851  0.0942  0.0814  0.1193  0.1029 |
| 6 | Streptodornase (Spy_49_1169) | DQDEPNIK  NFPDTTEILLGTK   QVVLQYVGIDENGDLLQIK | 957.5102  1447.8302  2143.2232 | +2  +2  +2 | 0.0699  0.0744  0.0708 |
| 7 | SpeB, Streptococcal, cysteine protease/Streptopain streptococcal pyrogenic exoprotein B (Spy_49_1690c) | NLFAAISTR   VGGHAFVIDGADGR   ELSQNQPVYYQGVGK  QYNWNNILPTYSGR  LDALNPSALGTGGGAGGFNGYQSAVVGIKP.- | 991.6132  1369.7638  1708.9380  1724.9188  2788.5667 | +2  +2  +2  +2  +3 | 0.0682  0.0900  0.0960  0.0918  0.1635 |
| 7 | CAMP factor (Spy_49_1010c) | VEALSDAIK  GTDIEATVNK   IIDPFASVDAIK   VINYPDLQPTDR   FNTETIYDFSSIGAR | 944.5856  1046.5914  1287.7856  1429.7952  1719.9048 | +2  +2  +2  +2  +2 | 0.0678  0.0670  0.0782  0.0751  0.0944 |
|  |  |  |  |  |  |

M, N and Q represent oxidation in methionine (M) and deamidation in asparagine (N) or glutamine (Q), respectively
