# Supplementary material for: CodY-mediated regulation of Streptococcus pyogenes exoproteins
Source: BMC Microbiol. 2012 Jun 21;12:114. doi: 10.1186/1471-2180-12-114 (PMC3438106; doi:10.1186/1471-2180-12-114)
Supplement: Additional file 3 — Table S3. Tandem mass spectrometry results of proteins excised from 2-DE gel (Figure 3). [file 1471-2180-12-114-S3.docx]

**Table S3. Tandem mass spectrometry results of proteins excised from 2-DE gel (Fig. 3).**

| **Spot No.** | **Spy_49**^a^ | **Name**^b^ | **MW** | **pI** | **Peptide matches**^c^ | **Coverage %^d^** | **Score**^e^ |
| --- | --- | --- | --- | --- | --- | --- | --- |
| 7311 | 1010c | CAMP Factor, Cfa | 28479 | 6.71 | 10 | 39 | 727 |
| 8306 | 1010c | CAMP factor, Cfa | 28479 | 6.71 | 7 | 28 | 228 |
| 2411 | 1455 | Streptodornase, Spd-3 | 30133 | 5.01 | 4 | 14 | 120 |
| 8505 | 1690c | Pyrogenic exotoxin B, SpeB | 43249 | 8.58 | 20 | 62 | 2025 |
| 7505 | 1690c | Pyrogenic exotoxin B, SpeB | 43249 | 8.58 | 13 | 42 | 450 |
| 7512 | 1690c | Pyrogenic exotoxin B, SpeB | 43249 | 8.58 | 19 | 62 | 1421 |
| 8612 | 0549 | Zinc-binding protein AdcA precursor | 58474 | 8.36 | 15 | 27 | 368 |
| 7608 | 0549 | Zinc-binding protein AdcA precursor | 58474 | 8.36 | 14 | 25 | 412 |
| 7203 | 1692c | Streptodornase B/Mitogenic Factor 1, SdaB | 30646 | 9.17 | 6 | 27 | 652 |
| 6204 | 1692c | Streptodornase B/Mitogenic Factor 1, SdaB | 30646 | 9.17 | 6 | 25 | 416 |
| 5204 | 1692c | Streptodornase B/Mitogenic Factor 1, SdaB | 30646 | 9.17 | 5 | 22 | 237 |
| 8709 | 0811c | Extracellular hyaluronate lyase, HylA | 92579 | 6.16 | 14 | 20 | 609 |
| 8708 | 0811c | Extracellular hyaluronate lyase, Hyla | 92579 | 6.16 | 12 | 16 | 300 |
| 8610 | 0549 | Zinc-binding protein AdcA precursor | 58474 | 8.36 | 15 | 29 | 473 |
| 8611 | 0549 | Zinc-binding protein AadcA precursor | 58474 | 8.36 | 15 | 27 | 368 |

^a^ The ORF designation is based on annotation of the NZ131 complete genome sequence (25).

^b^ Function annotation were retrieved from NCBInr (<http://www.ncbi.nlm.nih.gov>),

^c^The number of matching peptides to the target protein

^d^ The percentage of the protein sequence that was observed by MS/MS analysis of the mapping.

^e^The threshold was set up by Mascot server (hhtp://ww.matrixscience.com) based in Mowse algorithm at the significance level P> 0.05 for random hit, using 95% confidence interval, the minimum score of 36 was used for peptide identification (indicates identity or extensive homology).
