# Supplementary material for: CodY-mediated regulation of Streptococcus pyogenes exoproteins
Source: BMC Microbiol. 2012 Jun 21;12:114. doi: 10.1186/1471-2180-12-114 (PMC3438106; doi:10.1186/1471-2180-12-114)
Supplement: Additional file 4 — Table S4. Peptide characteristics used to identify proteins excised from 2-DE gel (Figure 3). [file 1471-2180-12-114-S4.docx]

**Table S4. Peptide characteristics used to identify proteins excised from 2-DE gel (Fig. 3).**

| Spot # | Protein ID | Sequences | Parent mass | Charge State | Error (Da) |
| --- | --- | --- | --- | --- | --- |
| 7311 | CAMP Factor (Spy_49_1010c) | AQVQEIK  VEALSDAIK  AIDAVDHMK  GTDIEATVNK  IIDPFASVDAIK  AIVFSTTQLTHK  VLGIKPFDVYNR  VINYPDLQPTDR  AIVFSTTQLTHKVEK  FNTETIYDFSSIGAR | 814.4946  944.5458  1014.5230  1046.5176  1287.7290  1344.7786  1419.8198  1429.7172  1700.9875  1719.8118 | +2  +2  +2  +2  +2  +2  +2  +2  +3  +2 | 0.9442  0.0280  0.0426  0.0068  0.0216  0.0385  0.0324  0.0029  0.0414  0.0014 |
| 8306 | CAMP Factor (Spy_49_1010c) | AQVQEIK  VEALSDAIK  AIDAVDHMK  GTDIEATVNK  IIDPFASVDAIK  VINYPDLQPTDR  FNTETIYDFSSIGAR | 814.4698  944.5468  1014.5144  1046.5554  1287.7534  1429.7532  1719.8526 | +2  +2  +2  +2  +2  +2  +2 | 0.0150  0.0290  0.0340  0.0310  0.0460  -0.9509  0.0422 |
| 2411 | Streptodornase (Spy_49_1455) | NLVTMTK  DQDEPNIK  NPLGMLYYENR  NFPDTTEILLGTK | 821.4682  957.4720  1384.6908  1447.7976 | +2  +2  +2  +2 | 0.0366  0.0317  0.0463  0.0418 |
| 8505 | Pyrogenic exotoxin B (Spy_49_1690c) | YHNYPNK   NLFAAISTR  SAEDIKLDK  QDWESQIDK  LDTTYAGTAEIK  EAQDSAITFIQK  VGGHAFVIDGADGR  KLDTTYAGTAEIK  ENFGYNQSVHQINR  ELSQNQPVYYQGVGK  NEKEAQDSAITFIQK  QYNWNNILPTYSGR  ENIASFMESYVEQIK  SPEILGYSTSGSFDVNGK  DYTYTLSSNNPYFNHPK  GIHYNQGNPYNLLTPVIEK  ENIASFMESYVEQIKENK  LDALNPSALGTGGGAGGFNGYQSAVVGIKP.-  QDWESQIDKELSQNQPVYYQGVGK  MAISELMADVGISVDMDYGPSSGSAGSSR | 934.4514  991.5626  1017.5490  1147.5308  1281.6566  1349.6982  1369.6906  1409.7364  1704.8120  1708.8548  1720.8770  1724.8270  1802.8512  1856.8842  2059.9402  2169.1327  2174.0383  2787.4144  3838.3757  2937.3022 | +2  +2  +2  +2  +2  +2  +2  +2  +2  +2  +2  +2  +2  +2  +2  +3  +3  +3  +3  +3 | 0.0218  0.0176  0.0148  0.0163  0.0114  0.0156  0.0168  -0.0037  0.0152  0.0128  0.0139  0.0000  0.0116  0.0050  0.0127  0.0108  0.0182  -0.0048  0.9544  0.0344 |
| 7505 | Pyrogenic exotoxin B (Spy_49_1690c) | YHNYPNK  NLFAAISTR   SAEDIKLDK  QDWESQIDK  LDTTYAGTAEIK  EAQDSAITFIQK  KLDTTYAGTAEIK  ELSQNQPVYYQGVGK  NEKEAQDSAITFIQK  ENIASFMESYVEQIK  SPEILGYSTSGSFDVNGK   LDALNPSALGTGGGAGGFNGYQSAVVGIKP.-  MAISELMADVGISVDMDYGPSSGSAGSSR | 934.4384  991.5488  1017.5446  1147.5384  1281.6466  1349.6792  1409.7370  1708.8412  1720.8484  1802.8286  1856.8596  2788.4250  2937.2449 | +2  +2  +2  +2  +2  +2  +2  +2  +3  +2  +2  +2  +3 | 0.0088  0.0038  0.0104  0.0239  0.0014  -0.0034  -0.0031  -0.0008  -0.0148  -0.0110  -0.0196  0.0219  -0.0229 |
| 7512 | Pyrogenic exotoxin B (Spy_49_1690c) | YHNYPNK  NLFAAISTR  SAEDIKLDK  LDTTYAGTAEIK  EAQDSAITFIQK  VGGHAFVIDGADGR  KLDTTYAGTAEIK  ENFGYNQSVHQINR  ELSQNQPVYYQGVGK  NEKEAQDSAITFIQK  QYNWNNILPTYSGR  ENIASFMESYVEQIK  SPEILGYSTSGSFDVNGK  DYTYTLSSNNPYFNHPK  GIHYNQGNPYNLLTPVIEK  ENIASFMESYVEQIKENK  LDALNPSALGTGGGAGGFNGYQSAVVGIKP.-  QDWESQIDKELSQNQPVYYQGVGK  MAISELMADVGISVDMDYGPSSGSAGSSR | 934.4326  991.5492  1017.5338  1281.6450  1349.6804  1369.6710  1409.7314  1704.7906  1708.8156  1720.8556  1725.7968  1802.8260  1856.8590  2059.9279  2169.1033  2174.0062  2787.3346  2837.2915  2937.2593 | +2  +2  +2  +2  +2  +2  +2  +3  +2  +3  +2  +2  +2  +3  +3  +3  +3  +3  +3 | 0.0030  0.0042  -0.0004  -0.0002  -0.0022  -0.0028  -0.0087  -0.0062  -0.0264  -0.0076  0.9698  -0.0136  -0.0202  0.0003  -0.0186  -0.0139  -0.0846  -1.0546  -0.0085 |
| 8612 | Zinc-binding protein adcA precursor (Spy_49_0549) | LTTDVAGK  TDVEQIK  ANAATYIEK  EIAQEINAH.-  SITVVENIR  TFTYTYAGK  AGQDYFTVMR  AAVLSPLEGLTEK  AGQDYFTVMRK  YIYFEENASSK  GVIGNDGDVSMLMK  YVQFSDHAIAPEK  VVTTFYPVYEFTK  FMFEAKEPNAGEFK  AAVLSPLEGLTEKEMK | 803.4588  831.4674  979.5248  1023.5222  1029.6084  1050.5270  1202.5640  1326.7644  1330.6578  1349.6318  1466.7000  1503.7696  1592.8322  1659.7594  1730.9185 | +2  +2  +2  +2  +2  +2  +2  +2  +2  +2  +2  +3  +2  +3  +3 | 0.0200  0.0337  -0.9566  0.0238  0.0266  0.0248  0.0250  0.0250  0.0239  0.0179  0.0255  0.0338  0.0196  -0.0009  0.0061 |
| 7608 | Zinc-binding protein adcA precursor (Spy_49_0549) | LTTDVAGK  ANAATYIEK   TVYNGYFK  EIAQEINAH.-  TFTYTYAGK  AYPEKAENFK  AGQDYFTVMR   AAVLSPLEGLTEK  AGQDYFTVMRK  YIYFEENASSK  GVIGNDGDVSMLMK   VVTTFYPVYEFTK   FMFEAKEPNAGEFK  AAVLSPLEGLTEKEMK | 802.4916  979.5258  990.5016  1023.5268  1050.5304  1195.6222  1202.5690  1326.7708  1330.6588  1349.6402  1466.7076  1592.8316  1659.8047  1730.9029 | +2  +2  +2  +2  +2  +2  +2  +2  +2  +2  +2  +2  +3  +3 | -0.9472  -0.9556  0.0206  0.0284  0.0282  0.0350  0.0300  0.0314  0.0249  0.0263  0.0331  0.0190  0.0444  -0.0095 |
| 7203 | Streptodornase B/Mitogenic Factor 1 (Spy_49_1692c) | VNAVTGTR  AGDILYSK  SHLIADSLGGDALR  TLGTSQITPALFPK   GTLTYANVEGSYGVR  AVVVSMQSSDNTINEK | 816.4716  865.4858  1423.7794  1472.8406  1585.7876  1736.8450 | +2  +2  +2  +2  +2  +2 | 0.0263  0.0313  0.0375  0.0168  0.0140  0.0200 |
| 6204 | Streptodornase B/Mitogenic Factor 1 (Spy_49_1692c) | VNAVTGTR  AGDILYSK  AQEWLEANR  TLGTSQITPALFPK  GTLTYANVEGSYGVR  AVVVSMQSSDNTINEK | 816.4742  865.4896  1115.5812  1472.8598  1585.8102  1736.8564 | +2  +2  +2  +2  +2  +2 | 0.0289  0.0351  0.0453  0.0360  0.0366  0.0314 |
| 5204 | Streptodornase B/Mitogenic Factor 1 (Spy_49_1692c) | VNAVTGTR  AGDILYSK  TLGTSQITPALFPK  GTLTYANVEGSYGVR  AVVVSMQSSDNTINEK | 816.4724  865.4726  1472.8336  1585.7832  1736.8316 | +2  +2  +2  +2  +2 | 0.0271  0.0181  0.0098  0.0096  0.0066 |
| 8709 | Extracellular hyaluronate lyase (Spy_49_0811c) | YTAPIEK  AYTTIEQR  IIFVGSNIK  ADDKSPEAIK  AEKDAQNIIK  QEFETYISK  KDDLEISDTIK  LDIDLLENNDK  ELLSDTSVPVQK  YAYMMLPNMTR  LDSYVASFNSMDK  FNAQSHVAGIEALR  TLVTQGNAFYNVYDNLK  TNYQQVGMTSLSDDAFVASK | 820.4598  980.5198  989.6160  1072.5542  1128.6368  1143.5684  1275.6766  1300.6524  1314.7252  1437.6324  1491.6764  1511.7946  1959.0008  2177.0212 | +2  +2  +2  +2  +2  +2  +2  +2  +2  +2  +2  +3  +2  +2 | 0.0268  0.0272  0.0251  0.0142  0.0230  0.0237  0.0209  0.0014  0.0222  0.0234  0.0213  0.0101  0.0271  0.0266 |
| 8708 | Extracellular hyaluronate lyase (Spy_49_0811c) | YTAPIEK  IIFVGSNIK  DYSAAANITK  AEKDAQNIIK  QEFETYISK  DDLEISDTIK  KDDLEISDTIK  ELLSDTSVPVQK  YAYMMLPNMTR  LDSYVASFNSMDK  TLVTQGNAFYNVYDNLK  TNYQQVGMTSLSDDAFVASK | 820.4710  989.6458  1052.5614  1128.6562  1143.5978  1147.6128  1275.7026  1314.7574  1437.6594  1491.7114  1959.0480  2177.0874 | +2  +2  +2  +2  +2  +2  +2  +2  +2  +2  +2  +2 | 0.0380  0.0549  0.0476  0.0424  0.0531  0.0520  0.0469  0.0544  0.0504  0.0563  0.0743  0.0928 |
| 8610 | Zinc-binding protein adcA precursor (Spy_49_0549) | LTTDVAGK  ANAATYIEK  TVYNGYFK  EIAQEINAH.-  TFTYTYAGK  KTFTYTYAGK  AGQDYFTVMR  AAVLSPLEGLTEK  YIYFEENASSK  GVIGNDGDVSMLMK  YVQFSDHAIAPEK  VVTTFYPVYEFTK  FMFEAKEPNAGEFK  AAVLSPLEGLTEKEMK  LKELDKDYTAALSDAK  MTAAEYKDYYTAGYK | 803.4698  979.5266  991.4942  1023.5262  1050.5306  1178.6256  1202.5710  1326.7712  1349.6408  1466.7058  1503.7738  1592.8562  1659.8197  1730.9371  1779.9742  1789.8355 | +2  +2  +2  +2  +2  +2  +2  +2  +2  +2  +3  +2  +3  +3  +3  +3 | 0.0310  0.0292  0.0292  0.0278  0.0284  0.0285  0.0320  0.0318  0.0269  0.0313  0.0380  0.0436  0.0594  0.0247  0.0488  0.0486 |
| 8611 | Zinc-binding protein adcA precursor (Spy_49_0549) | R.LTTDVAGK.E  K.TDVEQIK.I  K.ANAATYIEK.L  K.TVYNGYFK.D  R.EIAQEINAH.-  K.AYPEKAENFK.A  K.AGQDYFTVMR.K  K.AAVLSPLEGLTEK.E  K.AGQDYFTVMRK.N  K.YIYFEENASSK.V  K.KTMTFVRNGEK.K  K.GVIGNDGDVSMLMK.A  K.YVQFSDHAIAPEK.A  R.SITVVENIRDSLSK.A  K.VVTTFYPVYEFTK.G  R.FMFEAKEPNAGEFK.Y  K.AAVLSPLEGLTEKEMK.A  K.MTAAEYKDYYTAGYK.T | 803.4774  831.4686  979.5200  991.4988  1023.5222  1195.6188  1202.5648  1326.7660  1330.6634  1349.6394  1404.6372  1466.6986  1503.7435  1559.8357  1592.8452  1659.7966  1730.9470  1788.7543 | +2  +2  +2  +2  +2  +2  +2  +2  +2  +2  +2  +2  +2  +3  +2  +3  +3  +3 | 0.0386  0.0349  -0.9614  0.0338  0.0238  0.0316  0.0258  0.0266  0.0295  0.0255  -1.9892  0.0241  0.0077  -0.0162  0.0326  0.0363  0.0346  -1.0326 |
|  |  |  |  |  |  |

M, N and Q represent oxidation in methionine (M) and deamidation in asparagine (N) or glutamine (Q), respectively
